# Cardiovascular risk equations in type 1 diabetes: from generic models to personalized prediction

**DOI:** 10.1080/07853890.2026.2642404

**Published:** 2026-03-13

**Authors:** Tonet Serés-Noriega, Irene Aguilo-Lafarga, Verónica Perea, Clara Viñals, Antonio J. Amor

**Affiliations:** a Endocrinology and Nutrition, Centro Médico Milenium, Zaragoza, Spain; b Hospital Pharmacy Service, Hospital Universitario San Jorge, Huesca, Spain; c Endocrinology and Nutrition Department, Hospital Universitari Mútua de Terrassa, Terrassa, Spain; d Diabetes Unit, Endocrinology and Nutrition Department, Hospital Clínic, Barcelona, Spain; e Institut d’Investigacions Biomèdiques August Pi i Sunyer (IDIBAPS), Barcelona, Spain; f Centro de Investigación Biomédica en Red de Diabetes y Enfermedades Metabólicas Asociadas (CIBERDEM), Instituto de Salud Carlos III (ISCIII), Madrid, Spain; g Facultat de Medicina i Ciències de la Salut, Universitat de Barcelona, Barcelona, Spain

**Keywords:** Type 1 diabetes, cardiovascular risk, risk prediction, subclinical atherosclerosis, precision medicine, risk stratification

## Abstract

**Introduction:**

Cardiovascular disease (CVD) remains the leading cause of premature mortality in type 1 diabetes (T1D). Despite this burden, current prevention guidelines and risk stratification tools are largely extrapolated from type 2 diabetes or general populations, often failing to capture the unique pathophysiological drivers of atherosclerosis in T1D. This narrative review aims to critically evaluate current cardiovascular prevention strategies, identify the limitations of generic risk calculators, and analyse the clinical utility and discriminatory performance of population-specific risk equations.

**Discussion:**

Current clinical practice guidelines provide heterogeneous and inconsistent recommendations for T1D. Generic risk calculators (e.g. SCORE2, ACC/AHA pooled cohort equations) are limited by short-term prediction horizons and a lack of validation in T1D cohorts, frequently leading to poor diagnostic performances in this population. Conversely, T1D-specific equations, such as Steno Type 1 Risk Engine (Steno-Risk) and the emerging LIFE-T1D model, incorporate disease-specific variables such as diabetes duration or albuminuria. Available evidence indicates that T1D equations as Steno-Risk demonstrate superior discriminatory performance compared to generic scores identifying those individuals with higher cardiovascular risk. However, despite improved calibration, specific tools still classify a substantial proportion of patients with established subclinical atherosclerotic disease as ‘moderate risk,’ creating a grey area that complicates therapeutic decision-making.

**Conclusion:**

Generic cardiovascular risk algorithms are inadequate for the T1D population. While specific equations represent a significant advancement toward accurate stratification, mathematical models alone may be insufficient. Moving toward precision medicine requires a combined approach that integrates validated T1D-specific risk calculators with subclinical atherosclerosis screening (e.g. carotid ultrasound, CAC scoring) and novel biomarkers. This synergistic strategy is essential to refine classification in the ‘moderate risk’ category and guide individualised cardioprotective interventions.

## KEY MESSAGES

- Generic cardiovascular risk prediction tools are inadequate for individuals with type 1 diabetes (T1D), as they show poor predictive performances and fail to account for specific disease-related factors.
- Population-specific risk equations in T1D, such as Steno-Risk, demonstrate superior discriminatory performance identifying those with a higher cardiovascular risk compared to standard generic guideline risk calculators or classifications.
- To resolve the uncertainty in the ‘moderate-risk’ category, precision medicine in T1D requires a multi-modal approach that integrates validated specific equations with direct screening for subclinical atherosclerosis using imaging techniques.

## 1. Introduction

Cardiovascular disease (CVD) remains the leading cause of premature mortality in type 1 diabetes (T1D), reducing life expectancy by over a decade compared to the general population [1–4]. Despite improvements in clinical management, the rising global incidence of T1D implies a growing population at risk, exacerbated by early disease onset and prolonged exposure to the deleterious effects of the disease on the development and progression of atherosclerosis [5–7].

The pathophysiology of CVD differs between T1D and type 2 diabetes (T2D), although the specific underlying mechanisms remain incompletely understood [8]. While hyperglycemia triggers cardiovascular alterations in both conditions, T1D is uniquely characterized by the cumulative impact of early-onset hyperglycemia, glycemic variability, specific lipid metabolism alterations, and autoimmunity, among others [9]. In contrast, cardiovascular pathology in T2D is largely derived from the presence of multiple metabolic factors, such as insulin resistance and obesity [10]. Despite these distinct profiles, most recommendations regarding CVD prevention are based on extrapolations from T2D studies or older trials in which individuals with T1D were poorly represented. This represents a fundamental flaw, considering that the two conditions share little beyond hyperglycaemia. Furthermore, many RCTs assessing cardioprotective drugs (e.g. lipid-lowering agents, antiplatelets, antihypertensives) excluded individuals with T1D, leaving us without clear therapeutic targets for this population.

Given this context, several cardiovascular risk equations specifically designed for individuals with T1D have been developed, many of which are promising and critically needed for this vulnerable population. The aim of this narrative review is to outline the current cardiovascular prevention strategy in individuals with T1D, which is largely based on extrapolations and generic tools, as well as to analyse the main population-specific equations that incorporate additional parameters of significant prognostic value in this population.

## 2. Methods of searching

This article is a narrative review. Although a structured search was performed to identify relevant literature, no formal systematic selection process, PRISMA flow diagram, or risk-of-bias assessment was conducted. It focuses on evaluating current cardiovascular prevention strategies, identifying the limitations of generic risk calculators, and analysing the clinical utility and discriminatory performance of population-specific risk equations.

A comprehensive search of international PubMed and Embase (Elsevier, Amsterdam, The Netherlands) databases was conducted for all articles available up to 07 December 2025. The following search terms and Boolean operators were used in various combinations: (‘Diabetes Mellitus, Type 1’[MeSH Terms] OR ‘type 1 diabetes’[tiab]) AND (‘Cardiovascular Diseases’[MeSH Terms] OR ‘cardiovascular risk’[tiab] OR ‘cardiovascular prevention’[tiab]) AND (‘Risk Assessment’[MeSH Terms] OR ‘risk calculator’[tiab] OR ‘risk model’[tiab] OR ‘risk equation’[tiab] OR ‘risk prediction’[tiab]) AND (‘Atherosclerosis’[MeSH Terms] OR ‘subclinical atherosclerosis’[tiab] OR ‘carotid ultrasound’[tiab] OR ‘coronary artery calcium’[tiab] OR ‘CAC score’[tiab]). The suitability of articles collected from the electronic search was reviewed based on the abstracts. The search criteria were from lowest to highest specificity depending on the number of results available. We took all the results into account. We prioritised articles that evaluated clinical variables and were published in high-impact journals (top quartile of its discipline). Articles that were not related to the objective of the manuscript, conference abstracts, and duplicate articles were excluded from the review process. Only articles published in English and involving human subjects were considered.

## 3. Discussion

### 3.1. Current strategy for predicting cardiovascular events in individuals with T1D

The main clinical practice guidelines for cardiovascular prevention offer either strict or more flexible classifications and recommendations for cardiovascular risk (CVR) (e.g. initiating lipid-lowering treatment for a specific lipid target based on a defined risk level, or evaluating treatment based on fulfilling certain criteria). These guidelines recommend cardioprotective lifestyle changes (e.g. smoking cessation, weight control, heart-healthy diets such as the Mediterranean diet, physical activity) and the initiation of cardioprotective drugs in certain subgroups (e.g. acetylsalicylic acid, angiotensin-converting enzyme inhibitors [ACEi], angiotensin II receptor blockers, statins). Table 1 summarises the risk categorisations and lipid-lowering treatment recommendations from the most current and widely used guidelines. Most do not differentiate between diabetes types (T1D vs T2D); in others, some distinctions are made, though the therapeutic guidance is often unclear.

**Table 1.** Summary of cardiovascular risk categorisation and lipid-lowering treatment recommendations in people with type 1 diabetes according to main clinical practice guidelines.

Guideline	Risk category	Lipid-lowering treatment recommendations
American Diabetes Association (ADA) 2026 [86]	Age 20–39 years and ≥ 1 CVRF	Reasonable to initiate statin
	Age 40–75 years without ASCVD	Moderate-intensity statin
	Age 40–75 years and ≥ 1 CVRF	High-intensity statin for LDL-C goals <70 mg/dL and ≥50% reduction. Assess adding ezetimibe or PCSK9i if goal is not met, and BA if statin intolerant.
	Associated ASCVD	High-intensity statin for LDL-C goals <55 mg/dL and ≥50% reduction. Add ezetimibe or PCSK9i/inclisiran if goal is not met, and BA if statin intolerant.
	Age >75 years	Reasonable to initiate moderate-intensity statin and to continue statin treatment if already on it.
European Society of Cardiology (ESC) and European Association for the Study of Diabetes (EASD) 2019 [53]	**Moderate:** Age <35 years with <10 years duration and without other CVRF	Statins may be considered in asymptomatic persons >30 years of age, for LDL-C goals <100 mg/dL
	**High:** ≥10 years duration without target organ damage and ≥1 CVRF	Treatment for LDL-C goals <70 mg/dL and ≥50% reduction
	**Very High:** Associated ASCVD or Target organ damage or >20 years duration and childhood-onset	Treatment for LDL-C goals <55 mg/dL and ≥50% reduction
ESC 2021 [11]	Age ≤40 years + target organ damage and/or LDL-C > 100 mg/dL	Statin use may be considered
	**Moderate:** Age >40 years + <10 years duration, without target organ damage or additional CVRF	–
	**High:** Age >40 years without ASCVD or severe target organ damage and not meeting moderate risk criteria	Treatment for LDL-C goals <100 mg/dL (start) and <70 mg/dL and ≥50% reduction (intensification)
	**Very High:** >40 years + ASCVD or severe target organ damage: eGFR <45 mL/min/1.73 m² eGFR 45–59 mL/min/1.73 m² + urine albumin/creatinine ratio 30–300 mg/g Urine albumin/creatinine ratio >300 mg/g Presence of microvascular disease in ≥3 territories	Treatment for LDL-C goals <70 mg/dL (start) and <55 mg/dL and ≥50% reduction (intensification)
ESC 2023 [25]	Age >40 years without ASCVD or Age < 40 years + (other CVRF or microvascular complications or calculated 10-year ASCVD risk ≥10%)	Statin use must be considered
American Association of Clinical Endocrinology (AACE) 2022 [87]	**Low and moderate:**	Not applicable in T1D
	**High:** <20 years duration with <2 CVRF and without target organ damage	Moderate-intensity statin at onset for LDL-C goals <100 mg/dL
	**Very High:** >20 years duration and age >40 years (without ASCVD or target organ damage) or ≥2 CVRF	High-intensity statin +/− ezetimibe or bempedoic acid for LDL-C goals <70 mg/dL
	**Extreme:** Associated ASCVD or severe target organ damage (urine albumin/creatinine ratio ≥300 mg/g or eGFR <45 mL/min/1.73 m² or ABI <0.9 or systolic or diastolic left ventricular dysfunction)	High-intensity statin +/− ezetimibe or bempedoic acid for LDL-C goals <55 mg/dL
Canadian Cardiovascular Society 2021 [88]	Age ≥30 years and ≥15 years duration or Age ≥40 years or Associated microvascular complications	Initiate statins if LDL-C ≥77 mg/dL (≥2 mmol/mol)
	Associated ASCVD	Initiate statins if LDL-C ≥70 mg/dL (≥1.8 mmol/mol)
American Heart Association (AHA) and American College of Cardiology (ACC) 2018 [89] and 2019 [45]	Age 20–39 years and: ≥20 years duration or urine albumin/creatinine ratio (≥30 mg/g) or eGFR <60 mL/min/1.73 m² or Retinopathy or Neuropathy or ABI <0.9	Reasonable to initiate statin
	Age 40–75 years without other CVRF	Initiate moderate-intensity statin
	Age 40–75 years and multiple CVRF or calculated 10-year ASCVD risk ≥20%	Initiate high-intensity statin to reduce LDL-C ≥50%
	Associated ASCVD	Initiate high-intensity statin to reduce LDL-C ≥50% and add other lipid-lowering agents if goals of <70 mg/dL are not achieved
The National Institute for Health and Care Excellence (NICE) 2023 [90]	Age 18–40 years and <10 years duration	Consider statins
	Age >40 years or >10 years duration or presence of DKD or Presence of other CVRF	Offer statins: start with atorvastatin 20 mg
	Associated ASCVD	Initiate treatment for LDL-C goals ≤77 mg/dL (≤2 mmol/mol)

ABI: ankle-brachial index; ASCVD: atherosclerotic cardiovascular disease; CKD: chronic kidney disease; CVRF: cardiovascular risk factors; DKD: diabetic kidney disease; eGFR: estimated glomerular filtration rate; LDL-C: LDL cholesterol; PCSK9: proprotein convertase subtilisin/kexin type 9 inhibitors; T1D: type 1 diabetes.

On the other hand, there are CVR estimation equations validated in the general population, such as the Systematic Coronary Risk Evaluation 2 (SCORE2), proposed by the European Society of Cardiology (ESC) [11], which is not applicable to individuals with diabetes. Subsequently, the SCORE2-Diabetes equation was developed, although it has only been validated in individuals with T2D [12]. Others, such as the pooled cohort equations of the ACC and AHA [13], include approximately 10% of subjects with diabetes, with a residual representation of individuals with T1D. Moreover, the majority of these models consider diabetes as a binary predictive variable (yes/no), without accounting for other potential CVR factors, such as glycaemic control or the presence of microangiopathic complications. In 2024, the Predicting Risk of CVD EVENTS (PREVENT) equation was published by the American Heart Association (AHA) [14], which was studied and validated in a similarly small percentage of individuals with diabetes, and introduced, as a novelty, the optional inclusion of glycated hemoglobin (HbA1c) and the urinary albumin-to-creatinine ratio in its calculation.

The fact that the risk estimation time horizon in most cases is ten years, and that calculations are not permitted for individuals under 30–40 years of age, presents a major limitation for its application in T1D. This is particularly relevant given that early age at diagnosis has been strongly associated with atherosclerotic CVD (ASCVD) [7], and that atherosclerotic plaque formation may begin in childhood and adolescence, promoted by the cumulative effects of T1D [15,16]. Furthermore, it has been observed that the use of equations not specifically developed for the T1D population may lead to underestimation of CHD risk [17].

Data from the largest observational studies indicate that, in addition to glycaemic control and the presence of diabetic kidney disease (DKD), the main CVRFs in T1D are: tobacco use, duration of diabetes, systolic blood pressure, serum low-density lipoprotein cholesterol (LDL-C) levels, ACEi use, and a family history of premature CVD [18–21]. The loss of cardiovascular protection typically afforded by the female sex, observed in the general population, is also noted [22,23]. In this context, a systematic review and meta-analysis of observational studies involving a sample of nearly 200,000 participants estimated that the excess CVD risk was almost twice as high in women compared to men with T1D [23]. Furthermore, a recent Mendelian randomisation study demonstrated a causal relationship between T1D and atherosclerosis, suggesting the existence of CVR factors intrinsic to the disease itself [24].

In light of this predominantly observational evidence, some guidelines (Table 1) recommend a more aggressive therapeutic approach when certain CVR factors are present. Recently, the ESC produced a cardiovascular prevention guideline specifically for individuals with diabetes [25]. This document highlights the scarcity of RCTs in this population and offers cautious recommendations, suggesting early statin initiation in cases of long-standing T1D, the presence of two or more CVR factors, or a urinary albumin-to-creatinine ratio in the A2 range (30–300 mg/g). It also leaves open the possibility of using CVR estimation equations specific to the T1D population, as will be discussed in the subsequent section.

In summary, the current strategy for CVR prevention in T1D presents several limitations: (1) there are multiple clinical guidelines which are extensive, complex, and do not provide consistent classifications or recommendations; (2) the evidence in individuals with T1D is limited and predominantly observational in nature. This prevents the establishment of recommendations based on an adequate level of evidence and leads to extrapolation from studies conducted in T2D populations; (3) the main population-based CVR estimation equations have not been validated in individuals with T1D, are not applicable to younger individuals, and typically estimate risk over 5 or 10 years, which may result in underestimation of risk in individuals with T1D and a long disease duration.

Additionally, although no cardiovascular pharmacological intervention RCTs have been conducted specifically in people with T1D, data from a meta-analysis of RCTs carried out by the Cholesterol Treatment Trialists’ (CTT) Collaboration showed that statin therapy consistently reduced CVD risk in 18,686 individuals with diabetes [26]. The reduction in events observed in the T1D subgroup (*n* = 1466) was similar to that seen in individuals with T2D, although the low number of events did not allow for definitive conclusions. On the other hand, a retrospective cohort study of >11,000 individuals with T1D in primary prevention, using data from the Korean registry, demonstrated a 24% reduction in ASCVD risk among those treated with statins [27]. Further, a British emulation study in over 20,000 adults with T1D in primary prevention demonstrated that statin initiation was associated with significant reductions in all-cause mortality and major CVD events, specially in women, those with ≥40 years of age and patients with elevated LDL-C [28].

Finally, partly due to the limitations described above, uncertainty regarding therapeutic management is evident in routine clinical practice. For example, the use of statins or other cardioprotective drugs, and achievement of therapeutic targets based on some of the existing guidelines, remain far below recommended levels [29–31].

### 3.2. Towards accurate and individualised risk prediction in T1D: risk equations

The 2013 European Society of Cardiology/European Association for the Study of Diabetes (ESC/EASD) cardiovascular prevention guidelines already recommended against the use of general risk equations in individuals with diabetes [32]. Several tools have been developed for individuals with T2D, such as the one proposed by the United Kingdom Prospective Diabetes Study (UKPDS) [33] and the British QRISK2 model [34]. The latter, updated annually, has included individuals with T1D since 2017, and continues to do so in the updated QRISK3 model [35]. In individuals with T1D, the development of predictive equations has been slower and began later (Table 2).

**Table 2.** Characteristics of the main cardiovascular risk estimation equations validated in the type 1 diabetes population and ordered chronologically.

Equation	Estimation	Population	Variables included	Results
The Pittsburgh CHD in Type 1 Diabetes Risk Model 2010 [36]	Ischaemic heart disease (death, fatal/non-fatal AMI, pathological Q waves on ECG).	Follow-up: 8 years Derivation: *n* = 603 (EDC, no CVD, age ∼27 years, 50.4% men, diabetes duration ∼19 years, HbA1c ∼10.4%) Validation: n = 2328 (EURODIAB, no CVD, mean age ∼32.2 years, 48.4% men, diabetes duration ∼14.3 years, HbA1c NR)	Men (4 predictors): Circulating leukocytes, urine albumin/creatinine ratio ≥ 30 mg/g, HDL-C, diabetes duration Women (5 predictors): Waist-to-hip ratio, non-HDL-C, systolic blood pressure, treatment for HTN, diabetes duration	10-year prediction: Derivation: C-statistic = 0.89 (men) and 0.84 (women) Validation: C-statistic = 0.77 (men) and 0.78 (women)
Cederholm J, et al., 5-year CVD risk model 2011 [91]	Fatal or non-fatal CVD: AMI, stroke, unstable angina, coronary revascularisation	Mean follow-up: 4.9 years Derivation: *n* = 3661 (Swedish NDR, 6.1% with prior CVD, mean age 44.6 years, 55.6% men, diabetes duration 28 years, HbA1c 7.9%) Validation: *n* = 4484 (Swedish NDR, 6.2% with prior CVD, mean age 44.6 years, 54.9% men, diabetes duration 28.1 years, HbA1c 8.1%)	8 predictors: Diabetes duration, age at T1D diagnosis, total cholesterol/HDL-C ratio, HbA1c, systolic blood pressure, smoking, urine albumin/creatinine ratio >300 mg/g, prior CVD	5-year prediction: Derivation: C-statistic = 0.83 Validation: C-statistic = 0.80
Soedamah-Muthu S, et al., Prognostic model 2014 [39]	Fatal or non-fatal AMI, pathological Q waves on ECG, fatal or non-fatal stroke, advanced CKD, amputations, blindness and all-cause mortality	Median follow-up: 7.3–8.1 years Derivation: n = 1973 (EURODIAB, no CVD, mean age 30.3 years, 52% men, diabetes duration 11.5 years, HbA1c 8.3%) Validation: *n* = 554 (original EDC, no CVD, mean age 28 years, 49% men, diabetes duration 18.6 years, HbA1c 9.1%) *n* = 324 (recent EDC, no CVD, mean age 36.2 years, 48% men, diabetes duration 26.4 years, HbA1c 8.4%) *n* = 2999 (FinnDiane, no CVD, mean age 37.3 years, 51% men, diabetes duration 19 years, HbA1c 8.4%) *n* = 580 (CACTI, no CVD, mean age 36.3 years, 45% men, diabetes duration 22.7 years, HbA1c 8%)	5 predictors: Age, HbA1c, waist-to-hip ratio, urine albumin/creatinine ratio, HDL-C	7-year prediction: Derivation: C-statistic = 0.74 Validation: C-statistic = 0.79 (original EDC); 0.74 (recent EDC); 0.82 (FinnDiane) and 0.73 (CACTI)
Steno Type 1 Risk Engine (Steno-Risk) 2016 [40]	Fatal or non-fatal CVD: ischaemic heart disease, ischaemic stroke, heart failure and PAD	Median follow-up: 6.8 years Derivation: *n* = 4306 (Denmark, no CVD, mean age 42.2 years, 54.2% men, diabetes duration 16.6 years, HbA1c 8.4%) Validation: *n* = 2119 (Denmark, no CVD, mean age 44.2 years, 57.7 % men, diabetes duration 13.6 years, HbA1c 8.1%)	10 predictors: Age, sex, diabetes duration, systolic blood pressure, LDL-C, HbA1c, urine albumin/creatinine ratio, eGFR, smoking, physical activity (>30 minutes daily)	5-year prediction: Derivation: C-statistic = 0.826 (95% CI, 0.807–0.845) Validation: C-statistic = 0.803 (95% CI, 0.767–0.839) ^a^10-year prediction:* Derivation: C-statistic = 0.818 (95% CI, 0.802–0.833)
QRISK3 2017 [35]	Fatal or non-fatal CVD event: ischaemic heart disease, ischaemic stroke, TIA	Median follow-up: 4.4 years (∼30% with ≥ 10 years) General population of England aged 25–84 years, without CVD or statin use Derivation (*n* = 7.89M): *n* = 21,677 with T1D (46% men) Validation (*n* = 2.67M): *n* = 7,283 with T1D (46% men)	21 predictors: Age, sex, ethnicity, deprivation, systolic blood pressure, BMI, total cholesterol/HDL-C ratio, smoking, family history of premature CVD, T1D, T2D, treatment for HTN, rheumatoid arthritis, atrial fibrillation, CKD, systolic pressure variability, migraine, corticosteroid treatment, systemic lupus erythematosus, atypical antipsychotic treatment, severe psychiatric pathology and erectile dysfunction	10-year prediction: Validation: Women: C-statistic = 0.823 (95% CI, 0.789–0.857) Men: C-statistic = 0.804 (95% CI, 0.776–0.832)
McGurnaghan S, et al., CVD risk prediction tool 2021 [42]	Hospital admission or death from: AMI, stroke, unstable angina, TIA, PAD, coronary/carotid/peripheral revascularisation or major amputation	Follow-up: ∼7–8 years^b^ Derivation: *n* = 27,527 (Scottish registry, no CVD, mean age 35 years, 57% men, diabetes duration 13 years, HbA1c 8.7%) Validation: *n* = 33,183 (Swedish NDR, no CVD, mean age 31.9 years, 54.6% men, diabetes duration 16.9 years, HbA1c 8%)	15 predictors: Age, sex, diabetes duration, deprivation, HbA1c, BMI, systolic blood pressure, total cholesterol/HDL-C ratio, eGFR, urine albumin/creatinine ratio, presence of retinopathy, smoking, treatment for HTN, lipid-lowering treatment and atrial fibrillation	10-year prediction: Derivation: C-statistic = 0.82 (95% CI, 0.81–0.83) Validation: C-statistic = 0.85 (95% CI, 0.85–0.86)
LIFE-T1D model 2024 [46]	Non-fatal AMI, non-fatal stroke or cardiovascular death (including death from heart failure)	Follow-up: 8–12 years Derivation: *n* = 39,756 (Swedish NDR, no CVD, mean age 28 years, 55% men, diabetes duration 14 years, HbA1c 8.1%) Validation: *n* = 2709 (Denmark, no CVD, mean age 43 years, 58% men, diabetes duration 19 years, HbA1c 8.1%) *n* = 1022 (UK Biobank, no CVD, mean age 53 years, 56% men, diabetes duration 33 years, HbA1c 7.9%)	9 predictors: Age at diabetes diagnosis, smoking, BMI, systolic blood pressure, HbA1c, eGFR, non-HDL-C, urine albumin/creatinine ratio, presence of retinopathy	10-year prediction: Derivation: C-statistic = 0.846 (95% CI, 0.839–0.853) in men and 0.851 (95% CI, 0.843–0.859) in women Validation: Denmark, C-statistic = 0.767 (95% CI, 0.723–0.812) in men and 0.774 (95% CI, 0.716–0.833) in women UK Biobank, C-statistic = 0.711 (95% CI, 0.663–0.760) in men and 0.756 (95% CI, 0.692–0.819) in women
Bayesian Belief Network (BBN) [49]	Non-fatal AMI and stroke, cardiovascular death, confirmed angina, congestive heart failure, and coronary artery revascularization	Follow-up: 7 years Real data: *n* = 1293 (DCCT/EDIC, no CVD, mean age 42 years, 53% men, mean HbA1c 8.1%) Simulated data: *n* = 1600	9 risk factors: Age, sex, systolic blood pressure, HbA1c, LDL-C, HDL-C, smoking	Real data: C-statistic = 0.75 in the complete model and 0.76 in the missing model ^c^ Simulated data: C-statistic = 0.75 (95% CI 0.69–0.80) in the complete model and 0.71 (95% CI, 0.66-0.76) in the missing model^c^

AMI: Acute myocardial infarction; BMI: Body mass index; CACTI: Coronary Artery Calcification in Type 1 Diabetes; CI: Confidence interval; CKD: Chronic kidney disease; CVD: Cardiovascular disease; DCCT/EDIC: Diabetes Control and Complications Trial/Epidemiology of Diabetes Interventions and Complications; EDC: Pittsburgh Epidemiology of Diabetes Complications study; eGFR: Estimated glomerular filtration rate; EURODIAB: European Diabetes Prospective Complications Study; FinnDiane: Finnish Diabetic Nephropathy Study; HbA1c: Glycated haemoglobin; HDL-C: HDL cholesterol; HTN: Hypertension; LDL-C: LDL cholesterol; NDR: National Diabetes Register; NR: Not reported; PAD: Peripheral arterial disease; T1D: Type 1 diabetes; T2D: Type 2 diabetes; TIA: Transient ischaemic attack.

^a^The 10-year prediction was not carried out in the external validation cohort given that < 19% of the sample reached a follow-up of ≥10 years.

^b^Approximate calculation based on reported follow-up in person-years.

**^c^**when the method is applied in the presence of missing values, it is referred to as ‘missing model’. 5-fold cross-validation C-statistics showed similar values (data not shown).

One of the first attempts was carried out in 2010 on a small sample from the Pittsburgh Epidemiology of Diabetes Complication (EDC) cohort, composed of individuals with T1D diagnosed in childhood. It demonstrated adequate performance in predicting 10-year ischaemic heart disease risk [36]. A year later, based on the Swedish National Diabetes Register (Swedish NDR), an equation was developed and validated over a 5-year period in individuals both in primary and secondary prevention. Although it correctly discriminated ASCVD risk, it excluded individuals whose T1D diagnosis occurred at age 30 or older, accounting for up to one-third of people with T1D [37,38].

Subsequently, Soedamah-Muthu S et al. [39] published an equation derived from the European Diabetes Prospective Complications Study (EURODIAB) cohort, validated in samples from the FinnDiane study, the Coronary Artery Calcification in Type 1 Diabetes (CACTI) study, and two temporally distinct samples from the EDC. This ambitious study was conducted in a young and relatively healthy population, which resulted in a low number of observed events and may have reduced the performance of the proposed model. Furthermore, the outcome variables included all-cause mortality, amputations, and blindness (of any aetiology), based on the assumption of a shared pathophysiological origin, which is not necessarily valid.

Later, in Denmark, the Steno Type 1 Risk Engine (Steno-Risk) was developed [40]. This equation represents a significant advance in terms of practical use and clinical implementation for several reasons: (1) it was developed in a large, unselected sample representative of the T1D population; (2) it predicts traditional major adverse cardiovascular events (cardiovascular death, non-fatal myocardial infarction (MI), or stroke), and additionally includes heart failure and peripheral artery disease (PAD) events, the latter being a common initial manifestation of ASCVD [41]; (3) it is the first and only tool to include physical activity as a predictive variable, although this was not collected in a standardised manner and was binary in nature.

However, it also has two major limitations. First, it was developed and validated in a population with over 90% Danish ancestry, so external validation in other settings is required before widespread implementation. Second, in the validation cohort, less than 19% of participants had follow-up data beyond ten years, meaning the model could only be validated over a five-year horizon.

Although not specifically designed for the T1D population, the QRISK3 Equation [35] was developed using data from over 21,000 individuals with T1D from a dataset comprising over seven million individuals in the United Kingdom, which allowed for the inclusion of up to 21 CVR predictors, resulting in a model with strong calibration and discriminatory power.

More recently, in 2021 [42], an equation was developed using a large cohort of young adults from the Scottish register (*n* = 27,527) and validated in the Swedish NDR cohort (*n* = 33,183). The outcome was cardiovascular death or hospitalisation, and the model demonstrated good 10-year discriminatory power in both cohorts.

As outlined, the vast majority of cardiovascular event prediction equations focus on the short to medium term (5–10 years). Considering that age is the main CVRF, most predictions classify younger individuals as having low or, at most, moderate CVR, despite the fact that they may already harbour atherosclerotic plaques [15,43,44]. Consequently, the cardiovascular prevention scientific community increasingly supports the concept of lifetime or long-term CVR as a more realistic perspective on the impact of ASCVD in individuals whose estimated life expectancy exceeds a ten-year horizon [11,45].

In this direction, the LIFE-T1D equation was published in 2024 [46], offering a first approximation of lifetime CVR estimation (up to age 90) based on various assumptions and, understandably, without the possibility of external validation of the lifetime prediction. The predicted outcomes were three-point major adverse cardiovascular events (non-fatal MI, non-fatal stroke, or cardiovascular death), using a cohort of nearly 40,000 individuals with T1D from the Swedish NDR, and validated in Danish and British populations. Predictive variables included: age at diabetes diagnosis, smoking status, body mass index (BMI), systolic blood pressure, HbA1c, estimated glomerular filtration rate (eGFR), non-high density lipoprotein (HDL) cholesterol levels, urinary albumin-to-creatinine ratio, and presence of retinopathy. The ten-year performance was good, especially in the original cohort. One of the key innovations compared to earlier models was the adjustment of predictions for competing risks of non-cardiovascular death. Failure to make this adjustment can lead to overestimation of actual ASCVD risk [47]. For instance, although the QRISK3 equation maintains overall good performance with or without adjustment for competing risks, it becomes less precise when applied without adjustment in individuals with higher non-cardiovascular mortality risk, such as older patients and those with multiple comorbidities [48].

Recently, Moro et al. [49] developed a Bayesian Belief Network (BBN) model, representing a methodological departure from conventional risk calculators that rely on Cox proportional hazards regression. The BBN approach was applied to a cohort of 1293 subjects from the Diabetes Control and Complications Trial/Epidemiology of Diabetes Interventions and Complications (DCCT/EDIC) database, incorporating seven risk factors including age, HbA1c, HDL-C, systolic blood pressure, sex, LDL-C, and smoking status. The authors conducted a comprehensive performance evaluation encompassing internal validation on the complete cohort, assessment with missing values (e.g. LDL-C and HDL-C), 5-fold cross-validation with 100 random replications, and a simulation study using a comparable sample sample size as the real data one. The model demonstrated good predictive performance in all the analyses, with only modest decrements when there were missing values. Crucially, the Bayesian framework enables the model to explicitly represent causal dependencies between risk factors through a directed acyclic graph structure (DAGs), allowing clinicians to compute conditional probabilities for any subset of factors. Unlike standard risk calculators which usually substitute population means for missing values, the Bayesian approach handles incomplete data by computing expected values from conditional distributions, maintaining reasonable predictive accuracy even with absent covariates.

Table 3 shows a hypothetical example of 10-year CVR estimation using various general and T1D-specific equations in three illustrative risk scenarios: low, moderate, and high. It is important to note that the results across these models are not directly comparable, as they predict different outcomes (e.g. the ACC/AHA equation and LIFE-T1D predict classic three-point major adverse cardiovascular events, whereas Steno-Risk also includes PAD, and the model proposed by McGurnaghan S et al. incorporates more subjective outcomes such as the need for coronary revascularisation). Furthermore, only some models offer cut-off points to categorise CVR, often arbitrarily, and it must be remembered that categorising a continuous variable always entails a loss of information.

**Table 3.** Practical example of 10-year cardiovascular risk estimated with various equations specific and non-specific to the type 1 diabetes population in primary prevention.

Equation	‘Low’^a^	‘Moderate’^b^	‘High’^c^
ACC/AHA [89]	0.8%	3%	31.2%
Steno-Risk [40]	5.2%	12.3%	50.1%
QRISK3 [35]	3.7%	12.2%	37.4%
McGurnaghan S, et al. [42]	4%	12%	38%
LIFE-T1D [46]	0.9%	2.2%	26.2%

^a^’**Low Risk’**: White female, age 40 years, no prior cardiovascular disease, diabetes duration 10 years, HbA1c 7% (assumed constant over time), total cholesterol 180 mg/dL, LDL-C 100 mg/dL, HDL-C 55 mg/dL, blood pressure 120/70 mmHg, never smoked, urine albumin/creatinine ratio 10 mg/g, body mass index 22 kg/m^2^, waist-to-hip ratio 0.7, physical activity >30 min daily, estimated glomerular filtration rate 95 mL/min/1.73 m^2^.

^b^’**Moderate Risk’**: White female, age 50 years, no prior cardiovascular disease, diabetes duration 15 years, HbA1c 7.3% (assumed constant over time), total cholesterol 200 mg/dL, LDL-C 120 mg/dL, HDL-C 50 mg/dL, blood pressure 135/80 mmHg, ex-smoker for 1 year, urine albumin/creatinine ratio 15 mg/g, body mass index 25 kg/m^2^, waist-to-hip ratio 0.9, physical activity <30 min daily, estimated glomerular filtration rate 90 mL/min/1.73 m^2^.

^c^’**High Risk’**: White male, age 55 years, no prior cardiovascular disease, diabetes duration 30 years, HbA1c 8% (assumed constant over time), total cholesterol 230 mg/dL, LDL-C 154 mg/dL, HDL-C 38 mg/dL, blood pressure 140/90 mmHg, smoking 20 pack-years, urine albumin/creatinine ratio 32 mg/g, body mass index 30 kg/m^2^, waist-to-hip ratio 1.1, no physical activity, estimated glomerular filtration rate 60 mL/min/1.73 m^2^.

Other parameters included in some equations were considered not present/negative (e.g. presence of other comorbidities or microvascular complications, family history of premature cardiovascular disease, concomitant treatments) or in terms of equality (e.g. deprivation quintile), in all scenarios.

Cardiovascular risk considered low is shaded in green, moderate in orange and high in red, based on the cut-off points proposed by each of the equations or, if absent, an approximation was considered: <10%: low; 11%–19%: moderate; ≥20%: high.

As shown, in the hypothetical ‘low risk’ scenario (a 40-year-old woman, without microvascular complications, optimal glycaemic control, normal weight…), and in the ‘high risk’ case (a 55-year-old man, with diabetic kidney disease, poor glycaemic control, obesity…), the predictions align appropriately, yielding ‘low’ and ‘high’ percentages, respectively. However, the true value of using such predictive models does not lie in classifying individuals at either end of the spectrum, who rarely present uncertainty in routine clinical practice, but rather in the ability to upstage or downstage those who represent the broad intermediate segment of the population. In this regard, considerable variability is observed in the estimated risk for the ‘moderate risk’ case (a 50-year-old woman, overweight, suboptimal glycaemic and lipid control…), a very common profile in everyday clinical settings.

The persistence of this substantial ‘grey area’ across various predictive models warrants a deeper theoretical examination. Fundamentally, this phenomenon arises from the intrinsic limitations of current risk algorithms, which are heavily reliant on chronological age as the dominant driver of risk calculation. In T1D, where the onset of atherosclerotic disease is accelerated and often uncoupled from chronological age due to decades of hyperglycemic exposure, these models frequently underestimate the true CVD risk. Consequently, individuals with a high burden of subclinical disease are statistically downstaged into the ‘moderate risk’ category. Furthermore, traditional regression models rely on standard metabolic variables (e.g. HbA1c, LDL-C) that fail to capture the complex, non-linear pathophysiological drivers of T1D, such as oxidative stress, glycemic variability, and autoimmune-mediated inflammation [9]. Reliance on mathematical stratification alone is therefore inadequate for this population. Consequently, the integration of phenotypic markers (e.g. CAC scoring or carotid ultrasound) could be useful to resolve this uncertainty and accurately reclassify these patients.

### 3.3. General tools for predicting cardiovascular events are not useful in individuals with type 1 diabetes

Among the population-specific risk equations for T1D, Steno-Risk [40] has undoubtedly been the most widely utilized and analyzed. Our team observed the poor agreement between the generic risk scale proposed by the ESC/EASD-2019 and the Steno-Risk [50]. In this regard, we observed that 60% of those classified as very high risk by ESC/EASD-2019 presented as low or moderate risk according to Steno-Risk. Moreover, we demonstrated the inability of the former, and the ability of the latter, to correctly identify individuals with the presence and/or greater burden of subclinical carotid atherosclerosis. Accordingly, with a carotid plaque prevalence above 40%, the general classification demonstrated very poor sensitivity and specificity (<60%) for ESC/EASD-2019s very high-risk category in detecting atherosclerosis [50]. Moreover, more than half of the participants classified as very high risk by ESC/EASD-2019 had no carotid plaques. In fact, when analysing discordant individuals (higher risk estimated by Steno-Risk than by ESC/EASD-2019), the specific equation correctly identified those with atherosclerosis. Additionally, the performance of the Steno-Risk was independent of the CVR estimated by ESC/EASD-2019, other lipid variables not included in the equation itself, and the use of cardioprotective treatments, reinforcing the potential of this tool in the adult T1D population representative of our setting.

In the same line, other studies have highlighted the limitations of generic risk scales in predicting cardiovascular events in this population. Nearly two decades ago, Zgibor JC et al. demonstrated the lack of calibration of two of the most widely used tools at the time, the non-specific UKPDS [33] and Framingham [51] equations, in predicting ischaemic heart disease in the EDC cohort [17]. The analysed EDC cohort sample, while representative of that era, is considered high or very high risk (childhood-onset T1D with diabetes duration >20 years, mean HbA1c >10%). In this context, the failure to consider the presence of microvascular complications or the early age of onset of cardiovascular events resulted in a substantial underestimation of actual CVR by both the UKPDS and Framingham models, particularly among those at highest risk [17].

Similarly, a study conducted in an unselected sample of adults with T1D (*n* = 575), showed poor concordance between the ESC/EAS 2019 scale [52] (which, for T1D, applies the same classification as ESC/EASD-2019 [53]) and the Steno-Risk [54]. Notably, among those under 35 years of age, 30% were classified as very high risk according to ESC/EAS 2019, while nearly all (99%) were reclassified as moderate risk according to Steno-Risk, highlighting the lower accuracy of the generic scale in younger individuals. Likewise, among individuals over 50, nearly 30% of those classified as very high risk by ESC/EAS 2019 were considered moderate risk by Steno-Risk [54].

In a second study by the same Italian group, the predictive performance of both tools was retrospectively analysed in *n* = 456 adults with T1D. After a mean follow-up of 8.5 years, there were 24 non-fatal cardiovascular events, mainly myocardial infarctions and coronary revascularisations. The ESC/EAS 2019 scale classified over 90% of those who experienced an event as very high risk, whereas the Steno-Risk correctly identified only about 40% [55]. However, 85% of individuals who did not experience events were accurately classified as low risk according to Steno-Risk, while the generic scale was unable to discriminate reliably (<15% classified as moderate risk). In this cohort, bearing in mind the limitations posed by the small number of events, the very high risk category of ESC/EAS 2019 showed high sensitivity but low specificity (92% and 64%, respectively), while the low/moderate risk category of Steno-Risk achieved better overall performance (sensitivity 88%, specificity 85%).

Another small study from northern Italy (*n* = 223) found a positive correlation between CVR estimated by Steno-Risk and maximum carotid intima-media thickness (cIMT), as well as a twentyfold higher prevalence of carotid plaques among those classified as high vs low risk by this Equation [56]. These associations were also observed with the presence of diabetic retinopathy and neuropathy, which is unsurprising given the close relationship between microvascular and macrovascular complications in individuals with T1D [57]. A further positive association between Steno-Risk-estimated risk and cardiovascular disease (CVD) was reported, although only three non-fatal events occurred, so conclusions must be considered exploratory [56].

Another critical consideration is the applicability of these models across diverse ethnic populations, given that the majority were derived from cohorts of predominantly European ancestry. For instance, the Steno-Risk was developed in a population with over 90% Danish background, raising concerns about its generalizability to other ethnic groups with potentially differing CVD risk profiles. Addressing this gap, *Paliares* et al. evaluated the performance of Steno-Risk in a young and ethnically diverse cohort [58]. Their findings indicated that the Steno-Risk yielded good discriminative performance for CVD prediction. Furthermore, the model showed robust calibration, with no statistically significant differences found between estimated and observed risks at 5 and 10 years. Subsequently, the same group assessed the predictive utility of two specific equations (Steno-Risk [40] and McGurnaghan [42]) in this population. They reported that, despite moderate agreement between the two tools, both exhibited good discrimination and calibration [59]. While these results suggest a degree of transferability, broader external validation studies in different healthcare systems and resource settings are required to determine if region-specific recalibration is necessary to ensure equitable risk prediction globally.

Importantly, a systematic review and meta-analysis of observational studies also provided preliminary data on the superior discriminatory performance of disease-specific CVR tools [60].

Despite the limited number of studies evaluating the predictive performance of CVR equations for cardiovascular events, some data using other surrogate markers of CVD support the use of disease-specific CVR equations. Previously, our group demonstrated a positive and progressive association between Steno-Risk-estimated risk and cIMT, as well as with both the presence and number of atherosclerotic plaques in individuals with T1D [61]. Furthermore, high risk according to Steno-Risk, more so than other CVRFs (e.g. active smoking, older age, or hypertension), was the factor most strongly associated with atherosclerotic burden (defined as the presence of two or more carotid plaques) [61].

Similarly, *Llauradó G* et al. evaluated 179 adults with T1D in primary prevention and observed a direct and significant relationship between arterial stiffness, assessed by aortic pulse wave velocity, and Steno-Risk-estimated CVR [62]. Another small European study yielded similar results (positive correlation with Steno-Risk-estimated CVR) when assessing arterial stiffness using the gold standard carotid–femoral pulse wave velocity, but found no significant differences according to ESC/EAS 2019 CVR classification [63].

Finally, an Australian study included 85 adults with T1D not receiving statin therapy [64]. With a mean age of 35 years, nearly 80% had a coronary artery calcium (CAC) score of 0 Agatston units. Most of those classified as high risk by Steno-Risk had a CAC score >0 (95%). However, only one-third of those classified as very high risk by ESC/EAS 2019 had a positive CAC score. Moreover, Steno-Risk demonstrated good diagnostic performance in detecting a positive CAC score [64].

Although the limited available evidence appears to favour the greater utility of the specific Steno-Risk, it is noteworthy that across several of the studies mentioned, between 40% and 50% of individuals with atherosclerosis or established CVD were classified as moderate risk by Steno-Risk [50,55,64]. Population-specific equations are a step toward precision medicine, but further research is needed to refine risk classification using biomarkers and imaging. Furthermore, external validation studies and head-to-head comparisons of these specific equations are necessary to ensure their reliability across diverse populations. These advancements are crucial to narrow the grey area known as ‘moderate risk’ which currently precludes clear therapeutic decision-making in individual cases. In this regard, the combined use of subclinical atherosclerosis screening techniques (e.g. vascular ultrasound or CAC scoring) and the use of other novel biomarkers (e.g. lipoprotein disturbances, obstetric complications, dietary patterns, glycaemic variability, and/or inflammatory-derived factors novel biomarkers) may be of great utility [65–77].

Despite these advances, significant knowledge gaps persist. Various biomarkers (e.g. C-reactive protein, lipoprotein(a)) and the presence of subclinical atherosclerosis on imaging are strongly associated with CVD [78–80]. In the asymptomatic general population, atherosclerosis screening by imaging predicts cardiovascular events, and visualizing these plaques has been shown to improve individual risk profiles [81]. Cost-effectiveness studies in the general population suggest a potential benefit for imaging or multimodal strategies. However, this benefit relies heavily on the baseline risk of the cohort, making the precise selection of the target population critical [82,83]. Furthermore, the potential harms of this strategy must be carefully considered, including overtreatment and radiation exposure from CAC scoring [84].

Crucially, current evidence in the general population is predominantly observational, and data specific to T1D is even more limited. While specific equations improve discrimination, their integration with novel biomarkers and imaging requires validation in large, diverse prospective cohorts to establish their incremental predictive value over standard clinical variables. Future research must determine the cost-effectiveness of implementing routine subclinical atherosclerosis screening specifically for asymptomatic individuals without clearly low or high calculated CVD risk. Additionally, while specific equations improve discrimination, their integration with novel biomarkers requires validation in large, diverse prospective cohorts to establish their incremental predictive value over standard clinical variables. Finally, head-to-head comparisons of these strategies are necessary to define the optimal screening intervals and therapeutic thresholds in this population. In this context, our group recently proposed a practical protocol for CVR assessment and management incorporating carotid ultrasound in those at primary prevention and at higher CVR (DKD or ≥40 years of age or >10 years of T1D duration and at least one additional CVRF). However, robust cost-effectiveness studies are still needed to support its widespread clinical implementation [85].

## 4. Conclusions

The development of population-specific risk equations represents a fundamental advancement in capturing the unique pathophysiology of T1D. These tools, most notably Steno-Risk, demonstrate superior performance compared to generic CVR classifications. However, mathematical models alone are insufficient. As evidenced by the literature, a substantial ‘grey area’ of patients classified as moderate risk remains, where therapeutic decision-making is often ambiguous and preventing the timely initiation of cardioprotective therapies.

**Figure 1.**
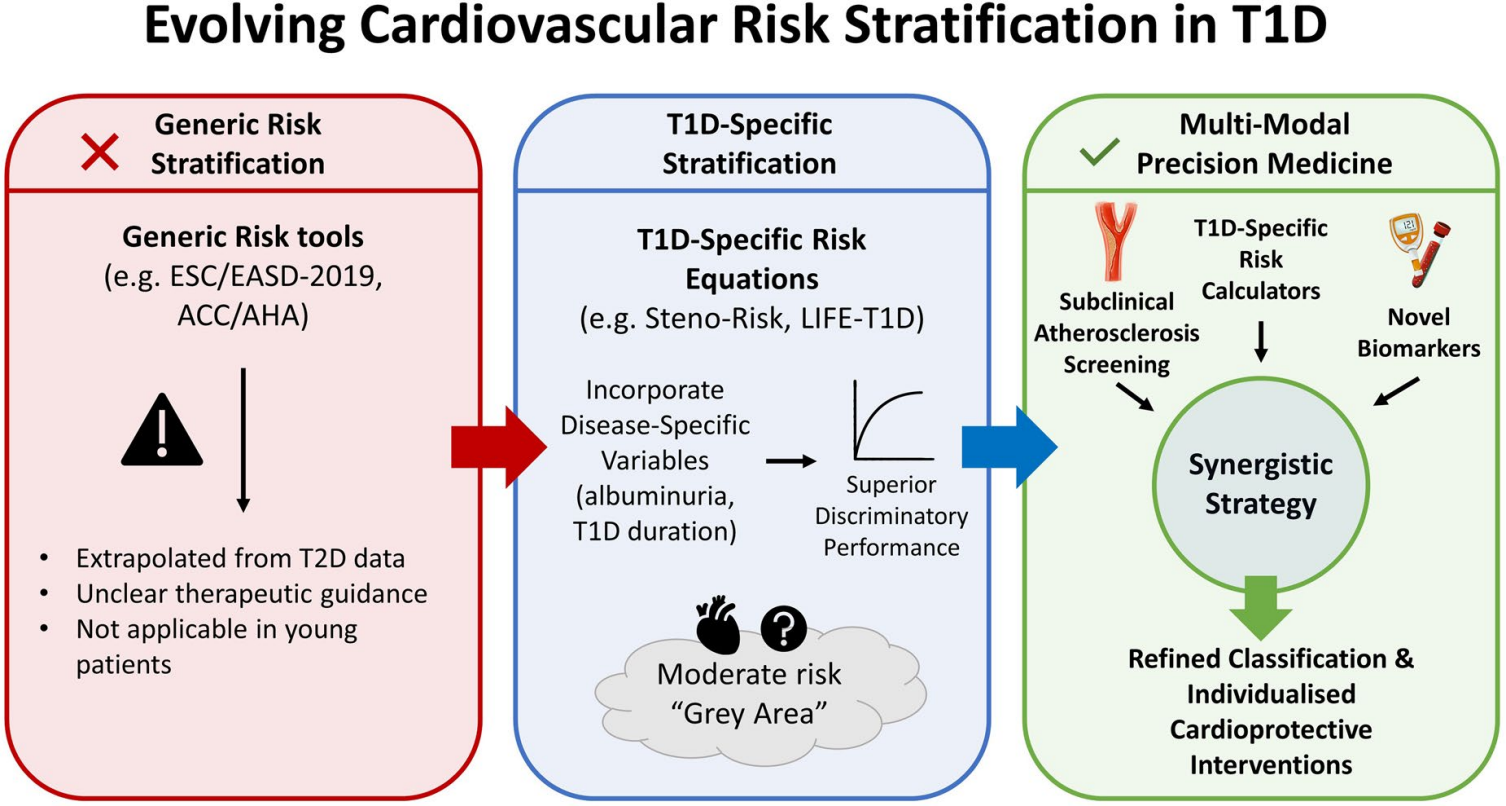
Evolving cardiovascular risk stratification in T1D: From generic models to personalized prediction. ACC/AHA: American College of Cardiology/American Heart Association; ESC/EASD: European Society of Cardiology/European Association for the Study of Diabetes; Steno-Risk: Steno Type 1 Risk Engine; T1D: Type 1 Diabetes; T2D: Type 2 Diabetes.

Consequently, the future of cardiovascular prevention in T1D must evolve from utilizing isolated risk estimation tools towards a comprehensive precision medicine strategy (Figure 1). It is crucial to distinguish this concept from traditional risk stratification, while the latter assigns a probabilistic risk based on population averages, precision medicine aims to characterize the individual’s unique phenotype in addition to their calculated probability. Specifically, we recommend that existing risk equations and assessment strategies be revised to further include imaging parameters (e.g. CAC score, carotid ultrasound) and novel biomarkers to enhance their predictive accuracy. By transitioning from statistical probability to the actual detection of preclinical disease presence, this synergistic approach allows clinicians to resolve the uncertainty surrounding the ‘moderate-risk’ category, ensuring that cardioprotective interventions are tailored to the specific biological reality of the patient rather than a generalized algorithm.

## Data Availability

Data sharing is not applicable to this article as no data were created or analysed in this research.
